# Jugular vein thrombosis due to Behçet disease

**DOI:** 10.11604/pamj.2015.20.216.6060

**Published:** 2015-03-10

**Authors:** Ahmed Belkouch, Lahcen Belyamani

**Affiliations:** 1 Emergency Departement, Mohammed V Military Hospital of Instruction, Rabat, Morocco

**Keywords:** Internal jugular vein thrombosis, behçet′s disease, CT angiography

## Image in medicine

A 46-years-old patient with a 7 years history of oral and genital recurrent ulceration with ocular redness since one year. He was admitted for the management of edema of the face and the neck, the onset was progressive without fever or poor general condition. On physical examination, the patient was aware, he had no fever, heart rate at 80b / min, blood pressure= 14/7, Spo2= 100%. There was no murmur on auscultation of the cervical vessels, and no neurologic deficit. Computed Tomography angiography was performed and revealed an almost complete thrombosis of the right internal jugular vein along 9 cm. the patient received curative dose anticoagulant therapy with good evolution. Behçet's disease is a multisystem disease characterized by a bipolar ulceration, skin lesions, and systemic vasculitis. The prevalence of vascular disease is about 25% and it alone represents the leading cause of death in this condition. Jugular thrombosis is an unusual event. Recent data from the literature support the beneficial effect of anticoagulation associated with corticosteroids and immunosuppressive therapy in its management.

**Figure 1:**
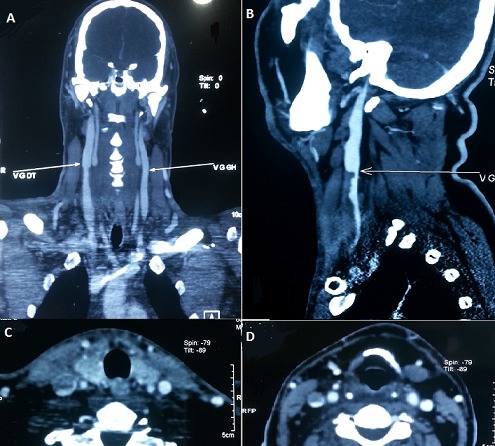
A: CT scan angiography in coronal cut showing the right internal jugular vein thrombosis; B: parasagittal cut showing the thrombosis; C and D: transverse cuts showing the same thrombosis

